# Analysis of uterine rupture at university teaching hospital Pakistan

**DOI:** 10.12669/pjms.314.7303

**Published:** 2015

**Authors:** Nousheen Aziz, Sajida Yousfani

**Affiliations:** 1 Dr. Nousheen Aziz, MS. Registrar, Department of Gynaecology & Obstetrics, Liaquat University of Medical & Health Sciences, Jamshoro, Sindh, Pakistan; 2 Dr. Sajida Yousfani, FCPS. Professor, Department of Gynaecology & Obstetrics, Liaquat University of Medical & Health Sciences, Jamshoro, Sindh, Pakistan

**Keywords:** Uterine Rupture, Scarred uterus, Oxytocin, Uterine repair, Hysterectomy

## Abstract

**Objective:**

To determine the risk factors, management modalities, fetomaternal outcome of uterine rupture cases at University teaching hospital in Pakistan.

**Methods:**

This retrospective descriptive study was conducted at the Department of Gynaecology and Obstetrics Liaquat University of Medical and Health Sciences (LUMHS) for a period of one year from January 1st to December 31st 2012. Main outcome measures were frequency, age, parity, booking status, risk factors, management modalities, fetal and maternal mortality associated with uterine rupture. The data was collected on pre-designed proforma analysed using SPSS Version 16 statistical package.

**Results:**

The frequency of ruptured uteri was calculated to be 0.67%, giving a ratio of 1:148 deliveries. Highest incidence was found in age group 25-30 (44.26%) with mean age of 30.36 years. and parity group 2-3 (57.37%) with mean parity 4.08. The risk factors for ruptured uterus include Caesarean section 43(70.49%), injudicious use of oxytocin 33(54.09%), obstructed labour 15 (24.59%) and multiparty 18 (29.50%). Repair of uterus was performed in 47(77.04%) cases. Maternal case fatality was 5(8.19%), while foetal wastage was 51 (83.60%).

**Conclusion:**

This study confirms the existence of a serious preventable obstetric problem, with significant maternal mortality and foetal wastage. Integrated efforts include Health education, focused antenatal care, skilled attendance, avoidance of injudicious use of oxytocin, and need of hospital based deliveries in patients with caesarean section which should be intensified to reduce this drastic obstetrical complication.

## INTRODUCTION

Uterine Ruture is a castrotrophic event with a significant effect on the reproductive function of the women and associated with high rates of fetal and maternal morbidity.^1^

In WHO systemic review of uterine rupture the incidence of rupture in general population was 5.3/10,000 birth. In Neitherland it was 5.9/10,000 deliveries.^2^,^3^ Although this unfortunate event is very rare in the developed world, has remained a major public health problem in developing countries, and often indicates poor obstetric care.^4^

Several direct and indirect factors have increased the risk of uterine rupture. These include wide spread poverty, illiteracy, cultural constrants^5^, advanced age, grand multiparity. Besides this, unskilled delivery and misuse of uterotonics pave the way to neglected and obstructed labour. The new and emerging cause responsible for majority of cases in modern obstetrics is previous caesarean scar rupture due to rising rate of caesarean delivery all over the world.^6^,^7^

This obstetric event is closely associated with maternal morbidities such as bladder rupture, vesicovaginal and rectovaginal fistula, foot drop and psychological trauma, anaemia and in long term because of surgical intervention the women may be sterilized which can lead to divorce and loss of economic support, showing that the magnitude of this obstetric complication is enormous.^8^

Uterine Ruture has the unique potential to impact negatively on Millennium Development Goals 4 and 5, that is to reduce perinatal and maternal mortality. This study aimed at establishing the incidence of uterine rupture, predisposing factors and changing trends in our areas, maternal and perinatal outcomes, management modalities at regional referred university hospital. Further review of this problem may be helpful in the development of appropriate preventive strategies, and to reduce such grave obstetrical complication.

## METHODS

A one year retrospective analysis of uterine rupture case records was performed at the Department of Gynaecology and Obstetrics Liaquat University hospital LUMHS from January 2012 to December 2012.

In this series, complete uterine rupture is defined as a full thickness separation of uterine wall and the overlying serosa. Cases of scar dehiscence constitutes separation of pre-existing scar that does not disrupt the overlying visceral peritoneum.

The clinical records of all patients were reviewed with regard to sociodemographic and reproductive parameters including age, parity, booking status, associated risk factors, management modalities, fetal and maternal mortality.

The surgical management comprised one of the three methods repair of uterus with tubal ligation, repair of uterus without tubal ligation, or hysterectomy. All patients were followed up until their discharge from hospital.

The data was collected on pre-designed proforma and analyzed by using SPSS Version 16 statistical package. Descriptive statistics were used for demographic data and summarized as means with standard deviation or frequency with percentage.

## RESULTS

The total maternity admissions were 10,004 with 9072 deliveries. The frequency of ruptured uteri was calculated to be 0.67%, giving a ratio of 1: 148 deliveries. Forty three (70.49%) of the patients had scarred uterus and eighteen (29.50%) had unscarred uterus giving a scarred to unscarred uterus ratio of 2.3:1. Out of them 48(78.68%) did not receive any antenatal care, while 13 (21.31%) patients were booked.

Mean age was 30.36±SD4.6 years, with the majority of cases 35(57.37%) aged 25-30 years. The mean parity was 4.08±2.8. Most of women were multipara or grand multipara 35 (57.37%), 18(29.50%) respectively. The gestational age of occurrence ranged from 26-40 weeks with a mean of 37±2.4 weeks. Table-I.

**Table-I:** Demographic characteristics.

Characteristic	No & %
*Age Of Patients*	
<25	3(4.91%)
25-30 years	35(57.37%)
31-35 years	18(29.50%)
36-40 years	05(8.19%)
*Parity*	
0-1	3(4.91%)
2-4	40(65.57%)
5&>5	18(29.50%)
*Period Of Gestation*	
<28 weeks	1(1.63%)
28-32 weeks	1(1.63%)
33-36 weeks	21(34.42%)
37 -40weeeks	38(62.29%)
Rural	42(68.52%)
Urban	19(31.14%)
Unbooked	48(78.68%)
Booked	13(21.31%)

The results are expressed in numbers and percentage.

The most important etiologic and predisposing factors were, injudicious use of oxytocin 33(54.09%), obstructed labour 15(24.59%), grand multiparty 18(29.50%), and previous uterine surgery due to caesarean section in 43(70.49%) of patients. Table-II.

**Table-II:** Risk and predisposing factors

Risk Factors	
Scarred Uterus	43(70.49%)
- Scarred uterus with augmentation with syntocinon	21(34.42%)
- Scarred uterus with spontaneous labour	20(32.78%)
- Scarred uterus with induction with prostin	2(3.72%)
Unscarred uterus	18(29.50%)
- Obstructed labour	15(24.59%)
- After coming head obstruction	2(3.27%)
- Fundal pressure	1(1.63%)
Grand Multiparity	18(29.50%)
Injudicious use of oxytocin	33(54.09%)

Majority of patients had a combination of risk/predisposing factors.

The uterine rupture was classified as complete in 32(52.45%) patients and incomplete in 29(47.54%) patients according to the surgical findings. fifty eight (95.08%) patients of the uterine rupture occurred outside the teaching hospital, mainly referred cases from primary 48(78.68%) and secondary health care levels or at local clinics 10(16.39%). Only three (4.91%) patients delivered at our centre.

Management was divided according to the type of rupture, parity and condition of the patient. Commonest surgical procedure carried out was uterine repair 47(77.04%), tubal ligation along with repair was done in 16(26.22%) patients. Hysterectomy was performed in 14(22.95%) cases. Two patients (3.27%) needed bladder repair along with hysterectomy.

All patients were anaemic, 18(29.50%) patients had severe anaemia (Hb<7g/dl), while 43(70.49%) patients were moderately anaemic (7-10g/dl). All patients needed blood transfusion as part of their intraoperative or postoperative management and mean blood volume transfused was 2.4±0.88 units. Only 8(13.1%) patient required single blood transfusion, while 28(45.90%), 17(27.86%), 8(13.11%) patients required two, three, and four units of blood transfusion respectively.

The average postoperative hospital stay was 8.49±2.99 (4-14) days. Majority 51(83.60%) stayed for 8-10 days in hospital, while 8(13.11%) patients stayed for 7 days and 2(3.27%) patients stayed for 14 days.

Maternal case fatality was 5(8.19%). Out of them two mothers died due to massive irreversible shock (one going in to DIC), while three patients died due to sepsis. The fetal outcome was poor. The total foetal wastage was 51 (83.60%). There were 34(66.66%) fresh intrauterine death (still birth), and 17(33.33%) old macerated intrauterine death giving overall perinatal mortality was 51 (83.60%). Live birth rate was 10(16.39%) only.

## DISCUSSION

Rupture Uterus (RU) is one of most devastating complication and is an index of failure of obstetric care at a point in time in a women reproductive carrier with dire fetomaternal consequences.^9^

Worldwide the reported incidence of this preventable complication has been varied from 2.6 to 0.006% depending upon obstetric services and standards provided in that region.^10^ In this study, the frequency of UR was 0.67% (1:148) which is comparable to that in developed country like Ireland is.02%^11^, which is much less than frequency reported in other developing countries like Nigeria (1:258)^12^, and in Ugenda (1:200).^13^

The incidence of UR in this study was contributed largely by unbooked cases which constituted about 78.68% of patients, as the institution is a referral centre, and received majority of patients in a serious condition which contributes to high mortality. The occurrences of uterine rupture among mostly unbooked patient have also been noted in other studies.^5^,^6^

The commonest age of occurrence of uterine rupture in our study was 25-30 years with a mean age 30.3±4.6 years, concordance with the mean age 30.8±-6.3 years reported Mbamara from Nigeria, while commonest age range of occurrence was 30-34 years.^14^ Multipaity has long been associated with uterine rupture, most (65.57%) of the affected patients in this study were in group para 2-4, while remaining cases (29.50%) were grand multipara, comparable to study conducted by Duhan reported that multiparity was predisposing factor in 97.9% of cases.^15^

Like in other studies scarred uterus, prolonged obstructed labour and injudicious use of oxytocics are the major risk factors identified in this study.^5^,^16^ This study however identified rupture to be more in patients with previously caesarean section(70.40%), as this is single most important factor in modern obstetrics. The noted scarred to unscarred uteri ratio of 2.3:1 is similar to 2.1:1 noted in Nigeria^14^ but a reverse of 1:1.7 noted in Afikpo.^10^ This may be due to rising rate of caesarean section in the country, Besides this inspite of counseling for institutional deliveries especially in case of previous caesarean section, tendency to seek unskilled intervension due to aversion for a repeat caesarean section, lack of proper surgical skills and aseptic techniques with poor settings by private practitioners leading to poor scar integrity.

In this study obstructed labour was the most common factor for rupture of uterus found in 15(24.59%) patients with non scarred uterus 18 (29.50%). Partograph is an important tool to detect deviation from normal labour, and early referral helps in prevention of such castrotrophe. Implementation of proper use of partograph in health setting at all levels is crucial.

In current study management of patients was dependant upon type and extent of uterine rupture, degree of haemorrhage, general condition of mother, mothers’ drive for future fertility. Uterine repair with or without tubal ligation performed in (77.04%) patients, may be justifiable because it was easiest and fastest procedure for surgeon. It may also be pointed that fair number of woman’s’ are in early part of reproductive carrier and wants to maintain their reproductive capability as loss of uterus is usually considered as loss of women hood in our society, with grave socio-cultural consequences and results in increased incidence of marriage breakdown with social trauma.

Hysterectomy was carried out in 22.95% of cases. Rupture uterus was noted as most common indication for emergency hysterectomy in many studies like Bushra and Shah N^17^,^18^ reported 34.86% and 35% respectively. Majority of these rupture uterus were caused by injudicious use of syntocinon. Similarly in this study oxytocin were administered injudiciously in (54.09%) cases by inexperienced, untrained and possible non medical personnel, which highlights the problems present in our society.

Illiteracy, ignorance, poverty, lack of antenatal care, poor access to maternal health services are all contributing factors. Besides this there is tremendious rise in private practitioners with minimal skill in the area as a result mishandling and delayed referral has resulted in increased morbidity and mortality. Restructuring the health system to ensure seamless communication between tertiary centres and the referring facilities is needed.

In our study all patients were anaemic and mean blood volume transfused was 2.4±8.8 units, while Mehmet^19^ reported that blood transfusion was necessitated in 42.62% of cases. That is probably due to the poor haemodynamic state in which our patents arrived and due to high prevalence of anaemia in pregnancy. Beside this the hospital stay is prolonged which has economic complication on loss of man hours and availability of hospital beds to those in need. In this study the average hospital stay was 8.49 days, while Mabamara^14^ reported that patients stayed in the hospital with a mean of 10.9 days.

In this study maternal case fatality of 5(8.19%) recorded is similar to coffie (9.8%).^20^ A good number of maternal deaths were recorded in the postoperative period and complication of overwhelming sepsis, irreversible shock, DIC and anaemia, were mainly responsible. Foetal mortality is even higher 83.60% in this study, this is in contrast to the observation in Netherland where over 90% of the fetuses were salvaged and there was no maternal death due to uterine rupture.^2^ The high maternal and fetal mortality figures are representative for available health care facilities, and calls for an integrated effort to prevent the cases.

This trend if not checked will jeopardize the realization millennium development goal (MGD) 4 and 5, which seek to reduce the maternal and perinatal mortality respectively. Widespread presence of obstetric care centres with adequate health facilities along with law regulating the practice of obstetric by defining who and where and minimum standards required should be regulated. As these measures will reduce if not eliminate the cases of rupture uterus.

The present study was conducted with objectives to find out the frequency of uterine rupture, predisposing factors and changing trends in our areas, maternal and perinatal outcomes, management modalities at regional referred university hospital. Further review of this problem may be helpful in the development of appropriate preventive strategies, and to reduce such grave obstetrical complication.

## CONCLUSION

This study reveals that there is still a high prevalence of rupture uterus. The observed risk factors for uterine rupture in this study are preventable. Although the etiological profile has changed from obstructed labour to scarred uterus which is now the leading cause of uterine rupture and can be reduced by decreasing the rate of caesarean section by proper selection of patients. Health education, Awareness about sign and symptoms of rupture uterus, high index of suspicion, councelling about need of timely booking and skilled attendance, importance of institutional deliveries especially of those with previous caesarean section, are important factors for reducing maternal morbidity and mortality associated with uterine rupture.
